# A novel *SPAST* gene mutation identified in a Chinese family with hereditary spastic paraplegia

**DOI:** 10.1186/s12881-020-01053-7

**Published:** 2020-06-03

**Authors:** Weiwei Yu, Haiqiang Jin, Jianwen Deng, Ding Nan, Yining Huang

**Affiliations:** Department of Neurology, Peking University First Hospital, 8 Xishiku Street Xicheng District, Beijing, 100034 China

**Keywords:** Hereditary spastic paraplegia, Gait disorder, Whole exome sequencing, *SPAST* gene, In-frame deletion

## Abstract

**Background:**

Hereditary spastic paraplegia is a heterogeneous group of clinically and genetically neurodegenerative diseases characterized by progressive gait disorder. Hereditary spastic paraplegia can be inherited in various ways, and all modes of inheritance are associated with multiple genes or loci. At present, more than 76 disease-causing loci have been identified in hereditary spastic paraplegia patients. Here, we report a novel mutation in *SPAST* gene associated with hereditary spastic paraplegia in a Chinese family, further enriching the hereditary spastic paraplegia spectrum.

**Methods:**

Whole genomic DNA was extracted from peripheral blood of the 15 subjects from a Chinese family using DNA Isolation Kit. The Whole Exome Sequencing of the proband was analyzed and the result was identified in the rest individuals. RaptorX prediction tool and Protein Variation Effect Analyzer were used to predict the effects of the mutation on protein tertiary structure and function.

**Results:**

Spastic paraplegia has been inherited across at least four generations in this family, during which only four HSP patients were alive. The results obtained by analyzing the Whole Exome Sequencing of the proband exhibited a novel disease-associated in-frame deletion in the *SPAST* gene, and this mutation also existed in the rest three HSP patients in this family. This in-frame deletion consists of three nucleotides deletion (c.1710_1712delGAA) within the exon 16, resulting in lysine deficiency at the position 570 of the protein (p.K570del). This novel mutation was also predicted to result in the synthesis of misfolded SPAST protein and have the deleterious effect on the function of SPAST protein.

**Conclusion:**

In this case, we reported a novel mutation in the known *SPAST* gene that segregated with HSP disease, which can be inherited in each generation. Simultaneously, this novel discovery significantly enriches the mutation spectrum, which provides an opportunity for further investigation of genetic pathogenesis of HSP.

## Background

Hereditary spastic paraplegia (HSP), also called familial spastic paraparesis or Stru ¨mpell-Lorrain disease, is a group of neurodegenerative and inherited heterogeneous neurological disorders characterized by a length-dependent distal axonal degeneration of the corticospinal tracts [1]. The progressive spasticity and pyramidal signs of the lower limbs are the prominent features of HSP, which can be well explained by the fact that the innervated function of the longest fibers toward to the lower extremity is prone to be affected [2].

According to whether accompanied by additional neurological or psychiatric symptoms such as ataxia, mental and cognitive changes, extrapyramidal signs, visual dysfunction or epilepsy, or with extra neurological signs, the diseases can be categorized into either pure HSP (pHSP) or complicated HSP (cHSP) [3, 4]. The onset age of HSP exhibits a wide range from childhood to over 70 years old depending on the underlying genetic defect, even in the family with a same mutation [5]. Therefore, it is hard to explain the interaction between the genotype and the phenotype of HSP.

Based on the distinguishably inherited trait, HSP can be classified into as autosomal dominant, autosomal recessive, X-linked, mitochondrial, or de novo [6]. Meanwhile, autosomal dominant HSP is the most common mode that accounts for approximately 70% of all HSP patients [7]. All modes of inheritance are associated with multiple genes or loci. Until now, there have been at least 76 spastic paraplegia associated with loci and more than 59 corresponding spastic paraplegia genes (SPG) have been identified [8]. The *SPG4/ SPAST* gene comprising 17 exons, identified as the 90-kb genomic region on chromosome 2 (2p22.3, [9], has been reported to be the most frequent cause of HSP and accounts for approximately 40% of pure autosomal dominant HSP and 10% of sporadic cases [10, 11]. Over 500 mutations have been identified in the *SPAST* gene. Generally, *SPAST* gene mutations have a tendency to cause pure HSP [12] and are more common in males than females [5]. Here, we report a novel *SPAST* gene mutation site (c.1710_1712delGAA) that presented in a Chinese family with HSP, significantly enriching the mutation spectrum of HSP gene.

## Methods

### Subjects

In this study, we recruited 15 subjects (female: male ratio is about 1:1; age range: 3–63 years) in total from a Chinese family with HSP. This family was enrolled in our study on the basis of the following criteria: (1) Based on Harding’s criteria [4], the proband, a 63-years-old female, was diagnosed with HSP; (2) The family of the proband had at least four affected relatives with HSP; (3) The family was potentially informative for designing a study to investigate the genetic mutations. Total of 15 individuals were performed with neurologic examination, four of which had the same clinical manifestations and the rest were all asymptomatic. The study was conducted according to the Declaration of Helsinki and was authorized by the Ethics Committee of Peking University First Hospital. The related written informed consents for publication of details and images were obtained from all the participants and the legal guardian of the patient aged 11 years in our study.

### DNA extraction

Whole genomic DNA was extracted from peripheral blood of the 15 family members using DNA Isolation Kit (Bioteke, AU1802) as previously described [13]. Concentrations of each DNA sample were measured on a Qubit fluorometer (Invitrogen, Q33216) using Qubit dsDNA HS Assay Kit (Invitrogen, Q32851). Meanwhile, 1% agarose gel electrophoresis was performed for quality control of each DNA sample.

### Libraries preparation and amplification

DNA libraries were established with KAPA Library Preparation Kit (Kapa Biosystems, KR0453) following the manufacturer’s instructions, which mainly contains three major procedures: end-repair of fragmented DNA, A-tailing, adapter ligation and amplification [14]. Purifications between procedures were achieved using Agencourt AMPure XP beads. After the ligation reaction with beads, 50 μl ligation was totally resuspended in 45 μl PEG/NaCl SPRI® Solution and then incubated at 37 °C for 2 min. Subsequently, the captured beads via a magnet were incubated until the liquid was clear. The beads were washed for three times using 200 μl 80% ethanol after discarding the clear supernatant and then was dried at room temperature (RT). Eventually, the beads captured on a magnet were thoroughly resuspended in 25 μl water and incubated for 2 min at RT until the liquid was clear to be proceed with library amplification.

Libraries amplification was fulfilled by polymerase chain reaction (PCR) under the following 25 μl reaction system: 12.5 μl 2× KAPA HiFi HotStart ReadyMix, 1 μl 5 μM each primer, 10 μl captured library beads suspension and 1.5 μl water. PCR amplification program was set up: 98 °C 2 min; 98 °C 30 s; 65 °C 30 s; 72 °C 30 s, 13 cycles; and a final step at 72 °C for 4 min. Subsequently, repeat the steps of washing and resuspending the beads as described above. The amplified libraries that were prepared for array capture were assessed with Qubit dsDNA HS Assay kit (Invitrogen, Q32851).

### Array capture and sequencing

Array capture was performed via the Agilent SureSelectXT2 Target Enrichment System as previously described [14]. Briefly, array hybridization was captured by mixing the pooled libraries with a buffer solution and oligo-blockers, which was incubated for 24 h at 65 °C. The hybridized library molecules were performed with Dynabeads® MyOne™ Streptavidin T1 (Invitrogen, #65601). The captured library was amplified as following: 21 μl 2× KAPA HiFi HotStart ReadyMix, 1 μl 5 μM primer, 20 μl captured library beads suspension. PCR amplification program was 98 °C 2 min; 98 °C 30 s; 65 °C 30 s; 72 °C 30 s, 13 cycles and a final step at 72 °C for 4 min. Purifications between procedures were conducted using Agencourt AMPure XP beads and the libraries were evaluated with Qubit dsDNA HS Assay kit (Invitrogen, Q32851). Finally, DNA libraries of the proband were analyzed by whole exome sequencing (WES). WES was carried out on the HiSeq2500 platform as paired-end 200-bp reads. Illumina Sequence Control Software (SCS) was used to evaluate the sequencing data, thus removing adapter sequences in the raw data and discarding low-quality sequencing reads. Conventional Sanger sequencing of the *SPAST* gene was further performed in 15 individuals from the Chinese family.

### In-silico predictions

Effects of the novel mutation on SPAST tertiary structure were predicted by RaptorX prediction tool [15]. Additionally, PROVEAN (Protein Variation Effect Analyzer) [16], a new algorithm, is also adopted to predict whether the mutation has an functional impact on the SPAST protein sequence variations.

## Results

### Clinical characteristics

**Fig. 1:**
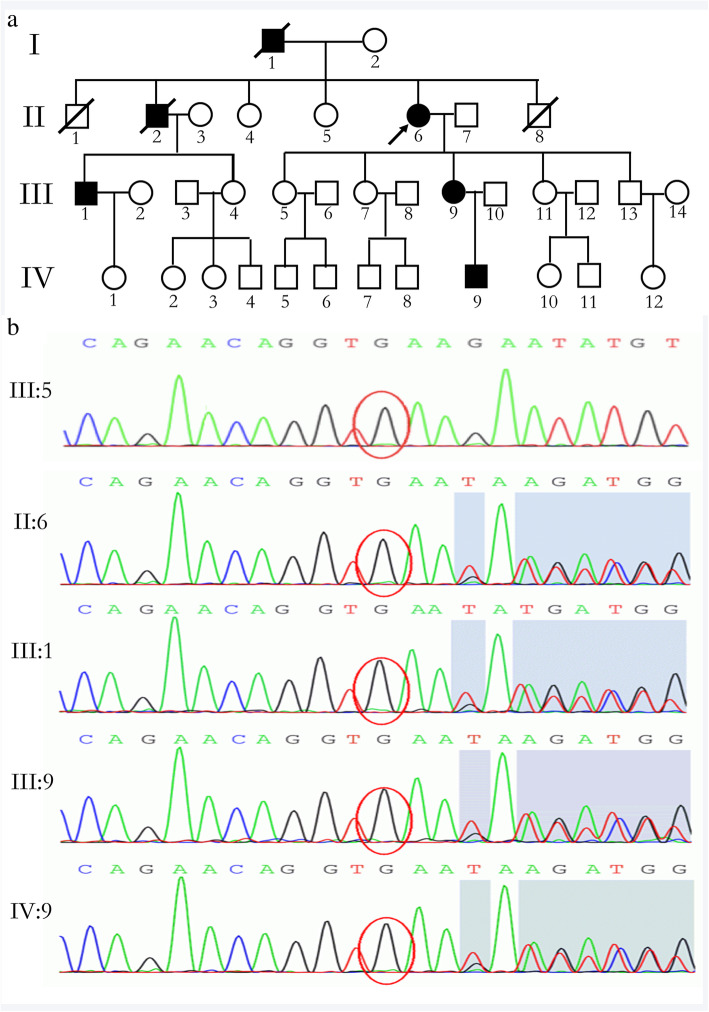
Pedigree of the investigated HSP family harbouring a novel SPAST gene mutation. **a**: The black arrow indicates the proband II:6. Squares indicate male, circles indicate females. Individuals affected with HSP are represented by black filled, while Healthy members are indicated by empty symbols. Slashes indicate already dead. **b**: Detection of the mutation of SPAST gene in a Chinese family. Sequence analysis revealed a newly identified in-frame deletion mutation in a heterozygous form in four affected individuals (II:2,III:1,III:9 and IV:9) within the family. The exon16 consists three nucleotides deletion(c.1710_1712delGAA). III:5 is the representative wild type sequences of the investigated healthy family members

The proband (II:2) (Fig. 1a), a 63-years-old female, presented to the outpatient clinic in our hospital due to her progressive difficulty walking caused by moderate spasticity of the lower limbs for 24 years. She has felt unknown gait disorder as early as in 1992. For the last 11 years, this gait disorder has gotten worse, especially in cold weather. And she was confined to a wheelchair at the age of 52. In the last year, the proband suffered from the frequently urged to urinating and bowel functions. Physical examination showed that she had brisk deep tendon reflexes in all four limbs, simultaneously accompanied with obvious corticospinal tract signs (Babinski^’^s signs was positive), and decreased sense of pain, light touch and vibration in the lower limbs characterized with stocking pattern-distributed sensory loss. Muscle strength of the upper limbs was normal, while both the extensors and flexors in the lower limbs were 3/5. The results obtained from routine laboratory tests, electromyography, cranial and cervical MRIs did not reveal any obvious pathognomonic alteration. For her previous history, the proband had ever received some treatment on rheumatoid arthritis because of the pain in both hips and knees 26 years ago, but the uncomfortable symptom was not getting better. 9 years ago, a traumatic injury on her back further aggravated her discomfort though the cranial and cervical MRIs were both normal at that time.

With regard to the proband’s family history (Fig. 1a), her parents were deceased, but her father had similar symptoms. In addition, she had three brothers and two sisters. One of her brothers who had similar symptoms has passed away and the rest were all asymptomatic. Details about her symptomatic father and brother are not clear. Physical examination gave the below results. III:9 aged 32 years had an abnormal spine physiological curvature. The shoulders of IV:9 aged 11 years were not equal. The (III:1) aged 46 years, III:9 and IV:9 showed brisk deep tendon reflexes in all four limbs and positive Babinski signs. All the three symptomatic patients all could not run and squat since young age. Despite their motor symptoms, the proband’s nephew(III:1) and the third daughter (III:9) have frequent urge to urinate and to have bowel functions at the age of 40 years and 27 years.

The proband’s the third daughter (III:9) and grandson(IV:9) suffer from other disease except HSP. The III:9 patient who was diagnosed with pulmonary hypertension has been suffering from chest tightness and shortness of breath for 3 years, losing the ability to work. Similarly, the echocardiography of IV:9 aged 11 years showed mild reflux at mitral valve and tricuspid valve.

Clinical features of the four affected individuals in the family have been summarized in Table 1, and their clinical commonalities and personalities are exhibited respectively. The four symptomatic patients have the different degrees of disability. The disability score was evaluated according to a four-point scale (1: normal, 2: able to walk but not run, 3: need the help of a walking aid or support, 4: walk on wheelchair) [17]. In addition, the onset age ranges from 3 to 30 years old, although they have the same mutation in the exon 16.

**Table 1:** Clinical features of the affected individuals within the family

Individual ID	II:6	III:1	III:9	IV:9
Sex	F	M	F	M
Age at onset (years)a	early 30s	early 10s	early 10s	3
Age at examination (years)	63	46	32	10
Disease duration (years)	> 33	> 36	> 22	7
Disability score b	4	2	2	2
Lower limb hyperreflexia	+	+	+	+
Lower limb spasticity	+	+	+	+
Lower limb pyramidal weakness	–	–	–	–
Babinski sign	+	+	+	+
Upper limb hyperreflexia	+	+	+	+
Upper limb spasticity	–	–	–	–
Sphincter disturbances	+	+	+	–
Scoliosis	–	–	+	–
Pes cavus	+	–	–	+
Sensory deficits	+	–	–	–
Mental retardation	–	–	–	–
concomitant diseases	–	–	Pulmonary hypertension	Reflux at mitral and tricuspid valve

+ indicates the presence of a feature, − indicates the absence of a feature, respectively

a: Age at onset was calculated approximately when appeared to have difficulty in walking first

b: Disability stages: 1: normal, 2: able to walk alone but not run, 3: need the help of a walking aid or support, 4: wheelchair user

### Genetic findings and prediction results of protein structure and function by different methods

The Exome Sequencing analysis of the proband exhibited a novel disease-associated mutation in exon 16 of the already known disease-associated *SPAST* gene, and the in-frame deletion was identified in the three affected family members (Fig. 1b II:2, III:1,III:9,IV:9). It is an in-frame deletion mutation in the heterozygous state: the GAA nucleotides deletion at codon 1710–1712 position and the circled nucleotide represents the codon 1710 position, where the mutation starts. (Fig. 1b II:2, III:1, III:9,IV:9). According to Human Gene Mutation Database (HGMDpro), the pathogenic mutation site c.1710_1712delGAA has not been reported until now. Therefore, it is a novel mutation. And there are no mutations were identified when analysis of other genes associated with HSP was performed: *PLP1, L1CAM, SPG11, SPG7, ATL1* and so on. While the rest asymptomatic family members had no mutations at this site (Fig. 1b III:5).

**Fig. 2:**
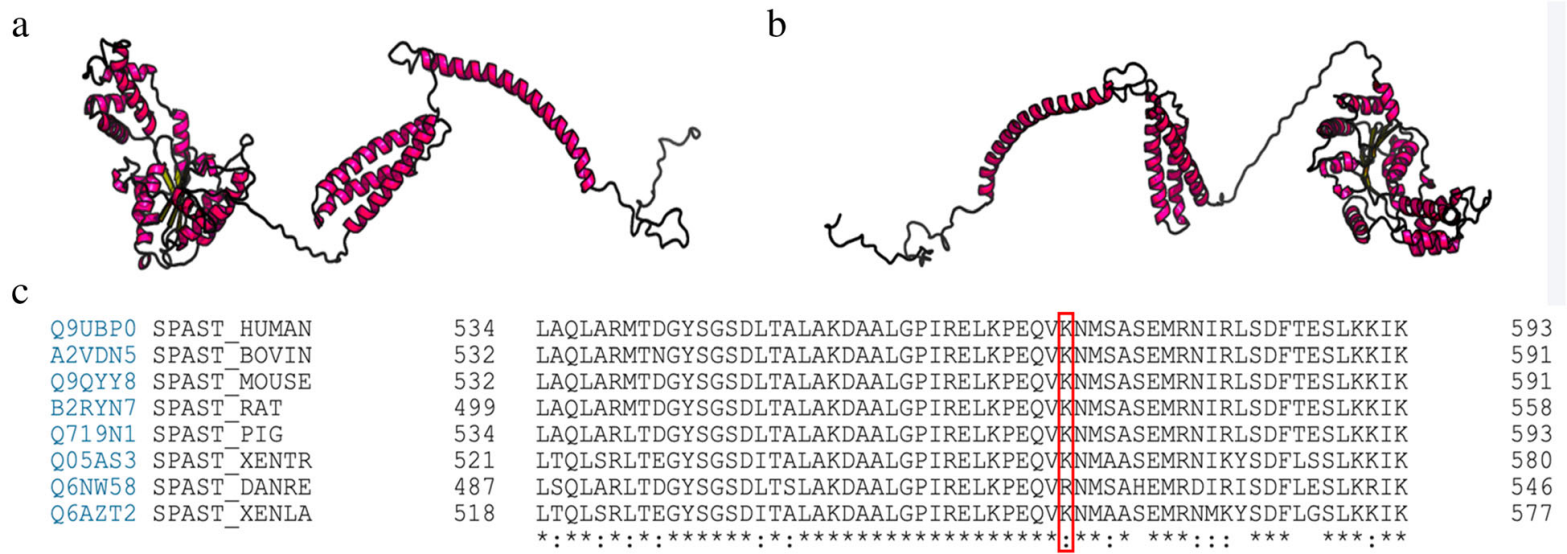
Tertiary structure alteration prediction of SPAST by RaptorX tool. **a**. The tertiary structure of native protein. **b**. Tertiary structure of p.K570del affected protein. The novel mutation we reported resulted in the synthesis of misfolded protein. **c**. The spastin protein sequence alignment across species showing the area of this in-frame amino acid deletion (red frame) and the surrounding residues

As highlighted in Fig. 1b, the results of RaptorX prediction showed that this new-found mutation we reported resulted in the synthesis of misfolded protein (Fig. 2b) in comparison to native one (Fig. 2a). We performed a protein sequence alignment across species showing the area of this in-frame amino acid deletion and the surrounding residues (https://www.uniprot.org/align/A20200502216DA2B77BFBD2E6699CA9B6D1C41EB2087CC0O). The result revealed that the spastin protein sequence across species is highly conserved at the position 570 of the protein (red frame) (Fig. 2c). Therefore, the lysine deficiency at the position 570 of the protein has a significant impact on the function. Additionally, the result of PROVEAN demonstrated that the mutation site c.1710_1712delGAA has an functional impact on the SPAST protein sequence variations. Given a list of genomic coordinates and variants (232,372,308,AGAA,A), the amino acid change(p.K570del) can be quickly determined and PROVEAN score is computed to be − 11.55, which is significantly lower than the score threshold (cutoff = − 2.5). The deletion variant was predicted as deleterious (Table 2). According to American College of Medical Genetics and Genomics (ACMG) criteria [18], we score this variant as likely pathogenic PM1, PM2, PM4, PP3.

**Table 2:**
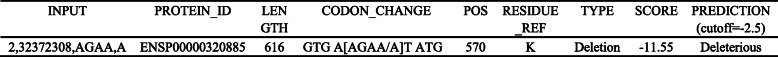
The genome variants results are represented as PROVEAN scores and predictions

## Discussion

According to the family history, accurate description of the clinical phenotype as well as cerebral and spinal MRI, we diagnosed the proband with HSP. Analyses of the Exome Sequencing revealed a novel disease-associated in-frame deletion in the *SPAST* gene. This mutation consists of three nucleotides deletions (c.1710_1712delGAA) within the exon 16. The onset age of patients in this family is highly variable, which is accordance with the previous study [19]. However, the onset age of affected individuals in our study is obviously earlier than previous studies [20], which promotes us to consider the existence of the new mutation site c.1710_1712delGAA. Additionally, affected individuals in our study presented clinical features of the pHSP, which is consistent with Orlacchio A’ s study [21].

The *SPAST* gene has 17 coding exons and encodes the protein spastin, a member of the AAA ATPase protein family. The protein spastin contains two main structural domains: the microtubule interacting and trafficking (MIT) domain in the N- terminus and the catalytic AAA domain at the C-terminus, in which the former mainly regulates microtubule organization and the later focus on ATPase activity associated with various cellular activities [22]. The two main domains are both essential to accomplish the main known function of spastin: microtubule (MT) severing [23]. More than 200 different mutations located in sites within the AAA region have been identified in patients with HSP-*SPG4* [24]. Therefore, it is believed that some mutated spastins may result in insufficient microtubule-severing activity by dominant-negative fashion. Additionally, another prevalent hypothesis is neurotoxicity of mutant spastin proteins. The *SPAST* gene presents two translation initiation codons, which allows to synthesis two spastin isoforms: a full-length isoform called M1(616 amino acid) and a slightly shorter isoform called M87(530 amino acid) that lacks the first 87 amino acid [25]. Studies on rodents show that M87 is more abundant in various tissues, whereas M1 is only appreciably detected in brain and spinal cord [26]. Besides that, axonal transport and neurite growth are not affected by the mutated M87 [27]. However, Mutant spastin proteins can form defective heterohexamers with wild-type (WT) spastin, and simultaneously produce toxic effect when presented as the tissue-specific M1 isoform [28, 29]. Even though M87 likely harbors the same AAA mutations as the M1 isoform, it is somehow degraded more effectively than mutated M1 in a dominant-negative scenario [30], thus possessing a lower toxicity.

## Conclusion

In Our study, a novel mutation in *SPAST* gene was found in a Chinese family with multiple affected family members, which significantly enrich the mutation spectrum of HSP. This novel mutation has been inherited at least four generations according to the related investigation, further emphasizing the closed interaction between the phenotypic and genetic heterogeneity of HSP. However, some shortcomings are still existed. For example, we haven’t performed functional laboratory studies about the novel mutation due to the unavailable patient cell lines and the related verification on which one molecular mechanisms above of *SPAST* gene mutants in our study cannot be realized. Haploinsufficiency is the prevalent mechanism at present. Using the haploinsufficiency model of HSP-SPG4 with a 50% decrease in active spastin levels has shown a lower MT severing [31, 32]. Another hypothesis is mainly that mutated spastin can form defective heterohexamers with wild-type (WT) spastin, and exert a dominant-negative effect. However, there is still considerable debate about the latter one hypothesis. It is unclear whether the mutant M87 can effectively impair the enzymatic activity of WT spastin [33]. Thus, further study should ascertain the role of causative genes to help better understand the relationship between genotypes and phenotypes.

## Data Availability

The datasets generated and/or analyzed during the current study are not publicly available in order to protect participant confidentiality.

## Abbreviations

HSP: Hereditary spastic paraplegia
SPG: Spastic paraplegia genes
CT: Computed tomography
MRI: Magnetic resonance imaging
SCS: Sequence Control Software
NGS: Next Generation Sequence
HGMDpro: Human Gene Mutation Database
MIT: Microtubule interacting and trafficking
MT: Microtubule
WT: Wild- type
ER: Reticulum membrane
CK2: Casein kinase 2
FAT: Fast axonal transport
PROVEAN: Protein Variation Effect Analyzer
ACMG: American College of Medical Genetics and Genomics
PCR: Polymerase chain reaction
RT: Room temperature

## References

[1] Depienne C, Stevanin G, Brice A, Durr A (2007). Hereditary spastic paraplegias: an update. Curr Opin Neurol.

[2] Esteves T, Durr A, Mundwiller E, Loureiro JL, Boutry M, Gonzalez MA, Gauthier J, El-Hachimi KH, Depienne C, Muriel MP (2014). Loss of association of REEP2 with membranes leads to hereditary spastic paraplegia. Am J Hum Genet.

[3] Lo Giudice T, Lombardi F, Santorelli FM, Kawarai T, Orlacchio A (2014). Hereditary spastic paraplegia: clinical-genetic characteristics and evolving molecular mechanisms. Exp Neurol.

[4] Harding AE (1983). Classification of the hereditary ataxias and paraplegias. Lancet (London, Engl).

[5] Proukakis C, Moore D, Labrum R, Wood NW, Houlden H (2011). Detection of novel mutations and review of published data suggests that hereditary spastic paraplegia caused by spastin (SPAST) mutations is found more often in males. J Neurol Sci.

[6] Finsterer J, Loscher W, Quasthoff S, Wanschitz J, Auer-Grumbach M, Stevanin G (2012). Hereditary spastic paraplegias with autosomal dominant, recessive, X-linked, or maternal trait of inheritance. J Neurol Sci.

[7] Fei QZ, Tang WG, Rong TY, Tang HD, Liu JR, Guo ZL, Fu Y, Xiao Q, Wang XJ, He SB (2011). Two novel mutations in the Spastin gene of Chinese patients with hereditary spastic paraplegia. Eur J Neurol.

[8] Parodi L, Fenu S, Stevanin G, Durr A (2017). Hereditary spastic paraplegia: more than an upper motor neuron disease. Rev Neurol (Paris).

[9] Hentati A, Pericak-Vance MA, Lennon F, Wasserman B, Hentati F, Juneja T, Angrist MH, Hung WY, Boustany RM, Bohlega S (1994). Linkage of a locus for autosomal dominant familial spastic paraplegia to chromosome 2p markers. Hum Mol Genet.

[10] de Bot ST, van den Elzen RT, Mensenkamp AR, Schelhaas HJ, Willemsen MA, Knoers NV, Kremer HP, van de Warrenburg BP, Scheffer H (2010). Hereditary spastic paraplegia due to SPAST mutations in 151 Dutch patients: new clinical aspects and 27 novel mutations. J Neurol Neurosurg Psychiatry.

[11] Erichsen AK, Inderhaug E, Mattingsdal M, Eiklid K, Tallaksen CM (2007). Seven novel mutations and four exon deletions in a collection of Norwegian patients with SPG4 hereditary spastic paraplegia. Eur J Neurol.

[12] de Bot ST, van de Warrenburg BP, Kremer HP, Willemsen MA (2010). Child neurology: hereditary spastic paraplegia in children. Neurology.

[13] Weiwie Y, Haiqiang J, Qian Y, Ding N, Yining H (2019). A novel PDCD10 gene mutation in cerebral cavernous malformations: a case report and review of the literature. J Pain Res.

[14] Ling X, Zhao d-h, Zhao J, Shen B, Yang X (2019). Episodic ataxia type 2 characterised by recurrent dizziness/vertigo: a report of four cases. Int J Neurosci.

[15] Scimone C, Donato L, Esposito T, Rinaldi C, D'Angelo R, Sidoti A (2017). A novel RLBP1 gene geographical area-related mutation present in a young patient with retinitis punctata albescens. Hum Genomics.

[16] Choi Y, Chan AP (2015). PROVEAN web server: a tool to predict the functional effect of amino acid substitutions and indels. Bioinformatics.

[17] McDermott CJ, Burness CE, Kirby J, Cox LE, Rao DG, Hewamadduma C, Sharrack B, Hadjivassiliou M, Chinnery PF, Dalton A (2006). Clinical features of hereditary spastic paraplegia due to spastin mutation. Neurology.

[18] Richards S, Aziz N, Bale S, Bick D, Das S, Gastier-Foster J, Grody WW, Hegde M, Lyon E, Spector E (2015). Standards and guidelines for the interpretation of sequence variants: a joint consensus recommendation of the American College of Medical Genetics and Genomics and the Association for Molecular Pathology. Genet Med.

[19] Durr A, Davoine CS, Paternotte C, von Fellenberg J, Cogilinicean S, Coutinho P, Lamy C, Bourgeois S, Prud'homme JF, Penet C (1996). Phenotype of autosomal dominant spastic paraplegia linked to chromosome 2. Brain.

[20] Orlacchio A, Kawarai T, Gaudiello F, Totaro A, Schillaci O, Stefani A, Floris R, St George-Hyslop PH, Sorbi S, Bernardi G (2005). Clinical and genetic study of a large SPG4 Italian family. Mov Disord.

[21] Orlacchio A, Patrono C, Borreca A, Babalini C, Bernardi G, Kawarai T (2008). Spastic paraplegia in Romania: high prevalence of SPG4 mutations. J Neurol Neurosurg Psychiatry.

[22] Salinas S, Carazo-Salas RE, Proukakis C, Schiavo G, Warner TT (2007). Spastin and microtubules: functions in health and disease. J Neurosci Res.

[23] White SR, Evans KJ, Lary J, Cole JL, Lauring B (2007). Recognition of C-terminal amino acids in tubulin by pore loops in Spastin is important for microtubule severing. J Cell Biol.

[24] Shoukier M, Neesen J, Sauter SM, Argyriou L, Doerwald N, Pantakani DV, Mannan AU (2009). Expansion of mutation spectrum, determination of mutation cluster regions and predictive structural classification of SPAST mutations in hereditary spastic paraplegia. Eur J Hum Genet.

[25] Claudiani P, Riano E, Errico A, Andolfi G, Rugarli EI (2005). Spastin subcellular localization is regulated through usage of different translation start sites and active export from the nucleus. Exp Cell Res.

[26] Solowska JM, Morfini G, Falnikar A, Himes BT, Brady ST, Huang D, Baas PW (2008). Quantitative and functional analyses of spastin in the nervous system: implications for hereditary spastic paraplegia. J Neurosci.

[27] Solowska JM, D'Rozario M, Jean DC, Davidson MW, Marenda DR, Baas PW (2014). Pathogenic mutation of spastin has gain-of-function effects on microtubule dynamics. J Neurosci.

[28] Eckert T, Le DT, Link S, Friedmann L, Woehlke G (2012). Spastin's microtubule-binding properties and comparison to katanin. PLoS One.

[29] Le DT, Eckert T, Woehlke G (2013). Computer simulation of assembly and co-operativity of hexameric AAA ATPases. PLoS One.

[30] Solowska JM, Garbern JY, Baas PW (2010). Evaluation of loss of function as an explanation for SPG4-based hereditary spastic paraplegia. Hum Mol Genet.

[31] Burger J, Fonknechten N, Hoeltzenbein M, Neumann L, Bratanoff E, Hazan J, Reis A (2000). Hereditary spastic paraplegia caused by mutations in the SPG4 gene. Eur J Hum Genet.

[32] Fonknechten N, Mavel D, Byrne P, Davoine CS, Cruaud C, Bonsch D, Samson D, Coutinho P, Hutchinson M, McMonagle P (2000). Spectrum of SPG4 mutations in autosomal dominant spastic paraplegia. Hum Mol Genet.

[33] Solowska JM, Baas PW (2015). Hereditary spastic paraplegia SPG4: what is known and not known about the disease. Brain.

